# Functional and biochemical characterization of the 20S proteasome in a yeast temperature-sensitive mutant, *rpt6-1*

**DOI:** 10.1186/1471-2091-9-20

**Published:** 2008-07-21

**Authors:** Aktar Uzzaman Chouduri, Toshinobu Tokumoto, Hideo Dohra, Takashi Ushimaru, Shinpei Yamada

**Affiliations:** 1 Department of Biology, Faculty of Science, National University Corporation, Shizuoka University, 836 Oya, Suruga-ku, Shizuoka 422-8529, Japan; 2 Institute for Genetic Research and Biotechnology, National University Corporation, Shizuoka University, 836 Oya, Suruga-ku, Shizuoka 422-8529, Japan

## Abstract

**Background:**

*Rpt6-1 *is a thermosensitive yeast mutant with a deletion of a gene encoding a regulatory subunit of the 26S proteasome, *RPT6*, which is able to grow at 25°C but not at 37°C. In this study, peptidase activities, activation profiles, and the subunit composition of the 20S proteasome purified from the *rpt6-1 *mutant was characterized.

**Results:**

The 20S proteasome purified from *rpt6-1 *exhibited low levels of peptidase activities in the absence of activators, but nearly same activated activities in the presence of activators, suggesting a gating defect in the proteasome channel. Detailed analyses of the composition of the 20S proteasome through separation of all subunits by two-dimensional gel electrophoresis followed by identification of each subunit using MALDI-TOF-MS revealed that two subunits, α1 and α7, differed from those of wild-type cells in both electrophoretic mobility and p*I *values. The changes in these two α-subunits were apparent at the permissive temperature, but disappeared during stress response at the restrictive temperature. Interestingly, upon disappearance of these changes, the levels of peptidase activity of the 20S proteasome in the *rpt6-1 *mutant were restored as the wild-type. These results suggest that two different forms of the α-subunits, α1 and α7, block the proteasome channel in the *rpt6-1 *mutant.

**Conclusion:**

Two α-subunits (α1 and α7) of the 20S proteasome in the *rpt6-1 *mutant differed from their wild-type counterparts and peptidase activities were found to be lower in the mutant than in the wild-type strain.

## Background

The 26S proteasome is an essential enzyme found in all eukaryotic cells [1,2] consisting of a cylinder-shaped multimeric complex, the 20S proteasome core particle, capped at each end by another multimeric complex, the 19S regulatory unit. The ATPase ring of the regulatory unit comprising six ATPase subunits (Rpt1-Rpt6) associates directly with the 20S proteasome and is thought to play multiple roles within the complex [3]. The 20S proteasome degrades unfolded substrate proteins in an ATP- and ubiquitin-independent manner [4]. It has been proposed that both unfolding and translocation of substrates are mediated by the six ATPases [5,6]. Crystallographic structural analysis shows that the ends of the 20S proteasome are in a closed state [7]. It has also been postulated that the ATPases can open the proteasome channel leading to the proteolytic chamber within the 20S proteasome [5]. The 20S proteasome functions as the catalytic center of the 26S proteasome that can cleave peptides on the carboxyl side of hydrophobic, basic, and acidic amino acids *in vitro *that have been designated as chymotrypsin-like, trypsin-like, and PGPH activities, respectively [8].

The entrance to the 20S proteasome cavity is closed [7]. The N-terminal tails of the different α-subunits adopt different conformations in the closed state and point inwards to block access through the proteasome channel [9]. Due to the narrow entrance of the 20S proteasome, access is restricted. It is thought that channel gating is regulated by the ATPase subunits in the 19S regulatory unit [10,11]. Little is known about this process in eukaryotic proteasomes. The closed channel of the eukaryotic 20S proteasome can be stimulated by a variety of means; one of which is mild chemical treatment, such as the exposure to low concentrations of SDS [12]. The stimulation may reflect opening of the proteasome channel that allows greater access of protein substrates to the proteolytic chamber. The N-terminal segments of various α-subunits that seal the central channel are generally rearranged to yield the activated form [7].

The *rpt6-1 Saccharomyces cerevisiae *mutant is temperature-sensitive for growth, being able to grow at 25°C but not at 37°C with cell division arrested in G2/Metaphase [13]. Normally, traffic through the ubiquitin-proteasome pathway is greatly increased in cells at an elevated temperature due to the abundance of misfolded or partially denatured protein substrates that must be removed. The defective proteasome in the *rpt6-1 *mutant is unable to process the increased protein traffic properly and cells eventually die due to the deleterious effects of accumulated misfolded proteins [14].

The *rpt6-1 *deletion mutant has been used previously [15-17]. In this study, we isolated the 20S proteasome from the *rpt6-1 *mutant and compared its peptidase activities to those of the wild-type strain. Surprisingly, distinct changes in peptidase activities and in subunit composition were observed in the 20S proteasome. A detailed comparative analysis using two-dimensional gel electrophoresis and mass spectrometry was then undertaken to investigate the molecular mechanism(s) responsible for the observed changes.

## Methods

### Chemicals

The peptide substrates, Suc-LLVY-MCA, Boc-LRR-MCA, and Z-LLE-MCA, were purchased from Peptide Institute (Osaka, Japan). The IPG DryStrip (pH 3–10 NL; non-linear) was purchased from Amersham Bioscience (Piscataway, NJ, USA). Acetonitrile (chromatogram-grade), acetone, iodoacetamide, urea, and TFA were from Wako Pure Chemical Industries (Osaka, Japan). Modified trypsin (sequencing grade) was from Promega (Tokyo, Japan). The CHCA (α-cyano-4-hydroxy-cinnamic acid) was obtained from Bruker Daltonics (Billerica, MA, USA). Acrylamide, bis-acrylamide, glycine, Tris, SDS, and DTT were from Nakalai Tesque (Kyoto, Japan). Other chemicals were domestic products (analytical grade).

### Yeast strains and growth

The following strains of yeast were used: the wild-type strain W303-1A (*MAT leu2 his3 trp1 ura3 ade2 can1*) and the *rpt6-1 *mutant (*MATα, ura3-52, leu2-Δ1, his3Δ-200, cim3-1*). The *rpt6-1 *strain was a gift from Dr. Akio Toh-e (Department of Biological Sciences, Tokyo University). Yeast cells were grown in YPAD medium (1% yeast extract, 2% meat peptone, 0.01% adenine, and 2% D(+)-glucose) at 25°C and 37°C to an absorbance of 2.0 at 600 nm (OD_600nm_).

### Plasmid construction

The expression plasmid p423GPD-RPT6 was constructed by insertion of the *RPT6 *ORF amplified by PCR using primers RPT6F1, 5'-**GTGGATCCCCCGGGCTGCAG**ATGACAGCTGCTGTAACATCC-3' and RPT6R1, 5'-**GTATCGATAAGCTTGATATC**TCACTTGAACAGCTTGGCG-3' (the bold bases are complementary to plasmid p423GPD), into plasmid p423GPD [18] digested with EcoRI. Plasmid construction was performed by homologous recombination in *rpt6-1 *cells according to standard methods.

### Purification of the 20S proteasome

The 20S proteasome was purified as described in [7] with minor modifications. Yeast cells were washed twice with ice-cold water and suspended in an equal volume of lysis buffer (40 mM Tris-HCl, pH 7.5, 2 mM EDTA, 1 mM DTT, and 20% glycerol). The cell suspension was subjected to five cycles of vigorous vortexing with glass beads (0.2 mm diameter) for 5 min. and chilling on ice for 1 min between cycles. After filtration, the cell lysate was centrifuged for 50 min at 96,000 × *g*. The supernatant was immediately applied to a Q-sepharose column (2.5 × 8 cm) previously equilibrated with 200 mM NaCl in buffer A (20 mM Tris-HCl, pH 7.5, 1 mM EDTA, 0.5% DTT, and 10% glycerol). The column was washed with the same buffer and eluted with a gradient of 200–600 mM NaCl in buffer A. Suc-LLVY-MCA hydrolyzing activity was assayed in all eluted fractions in the presence and absence of SDS. The proteasome eluted at ~400 mM NaCl. Active fractions were pooled and diluted three-fold with ice-cold water. The diluted sample was applied to a hydroxyapatite column (1.6 × 7.5 cm) previously equilibrated with buffer B (60 mM K-phosphate, pH 7.5, and 10% glycerol). The column was washed with the same buffer and eluted with a gradient of 60–300 mM K-phosphate containing 10% glycerol. The proteasome eluted at ~200 mM K-phosphate. Active fractions were combined and concentrated by ultrafiltration using an AMICON YM30 membrane (Millipore Corporation, USA). The concentrated sample was applied to a Superose-6 column (1.6 × 50 cm) and eluted with buffer A excluding DTT. The active fractions were combined, concentrated, and stored at -20°C prior to use. All preparative steps were carried out at 4°C. The concentration of the purified 20S proteasome was measured by the Bradford method [19] using BSA as a standard and purity was checked by nondenaturing-PAGE and SDS-PAGE.

### Assays of proteasome activity

The activity of the 20S proteasome to hydrolyze peptidyl substrates was measured at 37°C in a 200 μl reaction mixture containing 50 mM Tris-HCl, pH 7.6 and 8.5, 1 μg/ml 20S proteasome, 10 μM peptidyl substrate, various concentrations of SDS and linolenic acid, respectively for 1 h. The reaction was started by adding peptidyl substrate solution to the 20S-containing reaction mixture and was stopped by addition of 100 μl of 5% SDS. The reaction mixture was then diluted to 2.0 ml with 100 mM reaction buffer. Fluorescence readings of released 7-amido-4-methyl-coumarin (AMC) were measured at an excitation wavelength of 380 nm and an emission wavelength of 460 nm in a fluorescence spectrophotometer (F-3000, Hitachi, Tokyo). Activity was calculated as nmol of peptidyl substrate hydrolyzed per mg protein (20S proteasome) per min [20].

### Electrophoresis

SDS-PAGE was performed as described by Laemmli [21] using a 12% resolving gel and a 4% stacking gel. Silver staining was carried out by the 2D-Silver stain-II kit (Daiichi pure chemicals Co. Ltd., Tokyo) following the manufacturer's instructions. Nondenaturing-PAGE was performed similarly using a continuous 4% polyacrylamide gel without SDS in reagents and running buffer.

### Western blot analysis

Purified protein was subjected to SDS-PAGE in a 12% polyacrylamide gel and electrophoretically transferred to a PVDF membrane. Nonspecific binding was blocked with 5% nonfat dried skim milk (Wako) in Tris-buffered saline, 0.1% Tween-20 (TBS-T) overnight at 4°C. Blots were washed with TBS-T for 15 min., incubated with primary antibody in TBS-T for 1 h at room temperature. The primary anti-α7 monoclonal antibody (clone MCP72) was purchased from BIOMOL International, LP, USA and anti-*Xenopus *α1 polyclonal antibody was prepared as described in Wakata *et al*. [22]. After washing with TBS-T as above, the blots were incubated with horseradish peroxidase conjugated secondary antibody, mouse anti-rabbit IgG for the α7, and guinea pig anti-Xenopus IgG for the α1 subunit for 1 h at room temperature. The blots were washed as above and bound antibody was visualized using an ECL western blotting detection system [23].

### Two-dimensional gel electrophoresis

The purified 20S proteasome was subjected to two-dimensional polyacrylamide gel electrophoresis as described in Horiguchi *et al*. [24]. The purified 20S proteasome (70 μg) from each strain was mixed with 200 μl of IEF sample buffer containing 9 M urea, 2% nonidet P-40, 5% mercaptoethanol, and 1.5% pharmalyte (pH 3–10) and applied to IPG DryStrip (pH 3–10 NL; 130 × 3 × 0.5 mm). After rehydration for >20 h at room temperature, IEF was conducted in gradient mode for 10 min. at 0–500 V (first phase), 5 h at 500 V (second phase), 5 h at 500–3500 V (third phase) followed by 5 h at 3500 V (fourth phase) at 15°C on a Multiphor II flat bed electrophoresis unit (Pharmacia Biotech). After IEF separation, the gel strips were equilibrated for 10 min in equilibration buffer A containing 50 mM Tris-HCl, pH 6.8, 6 M urea, 30% glycerol, 1% SDS, and 0.25% DTT followed by equilibration buffer B for 10 min where DTT was replaced by 4.5% iodoacetamide. The second dimension of separation was carried out in SDS polyacrylamide vertical slab gels with 12% separating and 4% stacking gels and a constant current of 30 mA/gel. After electrophoresis, proteins were visualized by Coomassie Brilliant Blue R-250 staining and all visible spots were selected for in-gel digestion.

### In-gel digestion

The excised protein spots were destained by repeated washing with a 400 μl solution containing 50% acetonitrile and 50 mM NH_4_HCO_3_. The gel pieces were incubated with 100 μl of acetonitrile for 10 min and then dried under vacuum. The dried gel was incubated at 56°C with a 20 μl solution of 10 mM DTT and 50 mM NH_4_HCO_3 _for 45 min. and then dried again under vacuum. Digestions were performed overnight at 37°C with trypsin. The digested peptides were eluted by sonicating the gel pieces in a 3 μl solution of 50% ACN/1% TFA for 15 min in a mini sonicator. The peptide mixture was then transferred to a new tube, resuspended in 10 μl of 0.1% TFA, and used for sequence analysis.

### Peptide mass fingerprinting

ZipTip_μ-C18 _(Millipore) pipette tips were pre-wetted with a 10 μl solution of 50% acetonitrile/0.1% trifluoroacetic acid. Adsorption of peptides was performed by repeated pipetting of the peptide mixture solution from each gel spot. The resulting bound peptides were desalted with 10 μl of 0.1% TFA and spotted on a MALDI target plate that was double layered with α-cyano-4-hydroxycinnamic acid dissolved in acetone. The spots were air-dried and molecular mass information was obtained using a MALDI-TOF-MS Autoflex (Bruker Daltonics, Billerica, MA) in the positive ion mode. All MALDI-TOF-MS spectra were externally calibrated using a molecular weight standard mixture (Bruker Daltonics). The peptide MS fingerprint was subjected to a search of the NCBInr database using MASCOT software (Matrix Science, London, UK). The search was performed using the following settings: the *Saccharomyces cerevisiae *(Baker's yeast) database, trypsin digest, and variable modification-carbamidomethylation of cysteine. Initially, mass tolerances were set to ± 0.1 Da, and a single missed tryptic cleavage site was allowed. Mass tolerances were relaxed to ± 0.2 Da for PMFs which did not yield significant matches in the first search while all other parameters remained constant [25].

### Alkaline phosphatase treatment

The active Q-sepharose fraction (10 μg) was treated with alkaline phosphatase from calf-intestine (100 U, Boehringer Mannheim, Germany) in 50 mM Glycine-NaOH, pH 10 [26] or Tris-HCl, pH 7.6 [27] in the absence and presence of various concentrations of SDS at 37°C for 1 h. The alkaline phosphatase-treated 20S proteasome was then subjected to immunoblotting analysis using an anti-α7 monoclonal antibody.

### Data presentation

Peptidase activity values are presented as means and standard deviations for three independent sets of data.

## Results and Discussion

### Assay of 20S proteasome activity and activation profile

**Figure 1:**
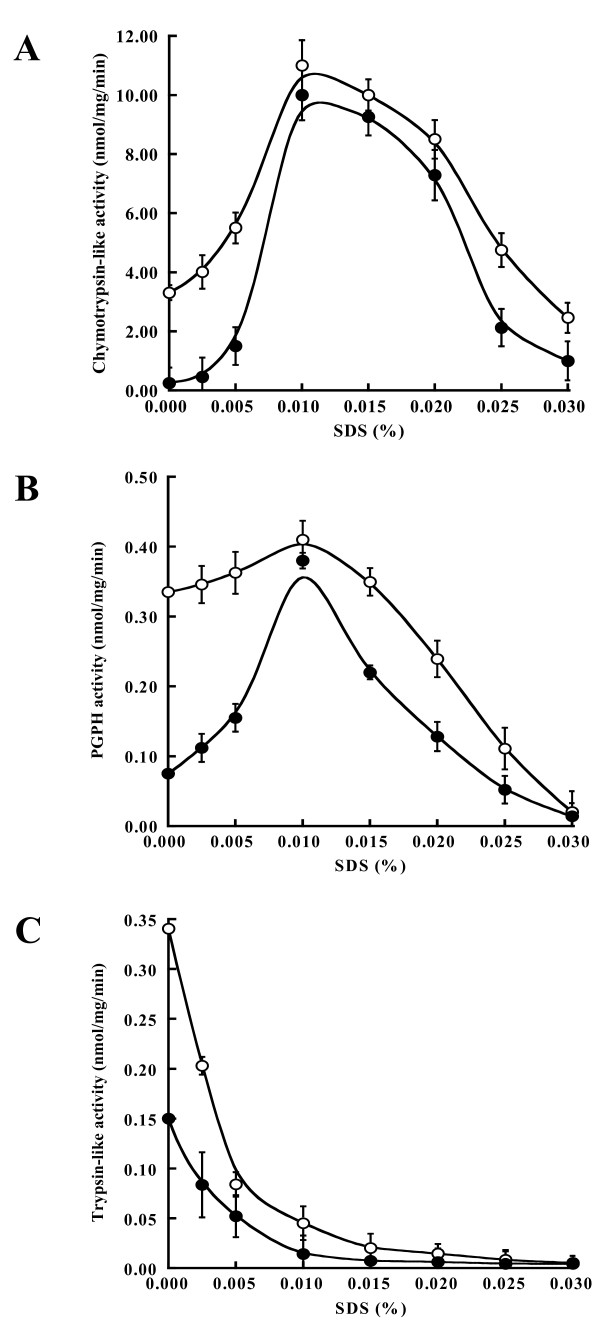
**SDS-mediated activation profile**. Peptide-hydrolyzing activity of the 20S proteasome was carried out in a 200 μl reaction mixture containing 50 mM Tris-HCl, pH 7.6, 1 μg/ml 20S proteasome, and 10 μM peptidyl substrates (**A**: Suc-LLVY-MCA for chymotrypsin-like activity; **B**: Z-LLE-MCA for PGPH activity; **C**: Boc-LRR-MCA for trypsin-like activity) and increasing concentrations of SDS at 37°C for 1 h. The reactions were started and stopped as described in methods. Symbols represent the wild-type strain (○) and the *rpt6-1 *mutant (●). Values are means ± SD of three independent experiments.

The comparative study of the 20S proteasome purified from the *rpt6-1 *mutant to that of the wild-type strain was carried out using enzymatic and proteomic approaches. In yeast, the 20S proteasome is auto-inhibited by the N-terminal segments of α-subunits in the center of the heptameric α-ring. The 20S proteasome is usually purified in a latent state having low levels of peptidase activities [28] that can be subsequently activated by a mild chaotropic agent, such as SDS or linolenic acid [29]. These agents selectively denature the N-terminal region and open the α-channel of the 20S proteasome cavity [30,31]. By use of an enzymatic approach, peptidase activities of the 20S proteasome purified from the *rpt6-1 *mutant (20S_r _proteasome) and activation profiles were compared to those of the wild-type strain towards three peptidyl substrates; Suc-LLVY-MCA for chymotrypsin-like activity (hereafter termed LLVY-activity), Z-LLE-MCA for PGPH activity (LLE-activity), and Boc-LRR-MCA for trypsin-like activity (LRR-activity). Relative to the 20S proteasome purified from the wild-type strain (20S_w _proteasome), 20S_r _proteasome exhibited weak peptidase activities in the absence of SDS (Fig. 1). Optimal activities were found at 0.01% SDS in both yeast strains as reported by others [8,32] and that seemed to reflect a convergence (Figs. 1A and 1B). The SDS appeared to inactivate LRR-activity as previously found by Thomas *et al*. (Fig. 1C) [33]. Trypsin-like activity and its SDS-mediated profile in the 20S_r _proteasome was evaluated using the peptidyl substrate Boc-LRR-MCA even though this type of peptidase is inactivated by SDS. In the absence of SDS, the 20S_r _proteasome hydrolyzed less peptidyl substrate than the 20S_w _proteasome, but both were almost equally active in its presence. This result suggests that the entrance of the α-channel in the 20S_r _proteasome is too narrow to allow passage of a peptidyl substrate to the catalytic chamber.

**Figure 2:**
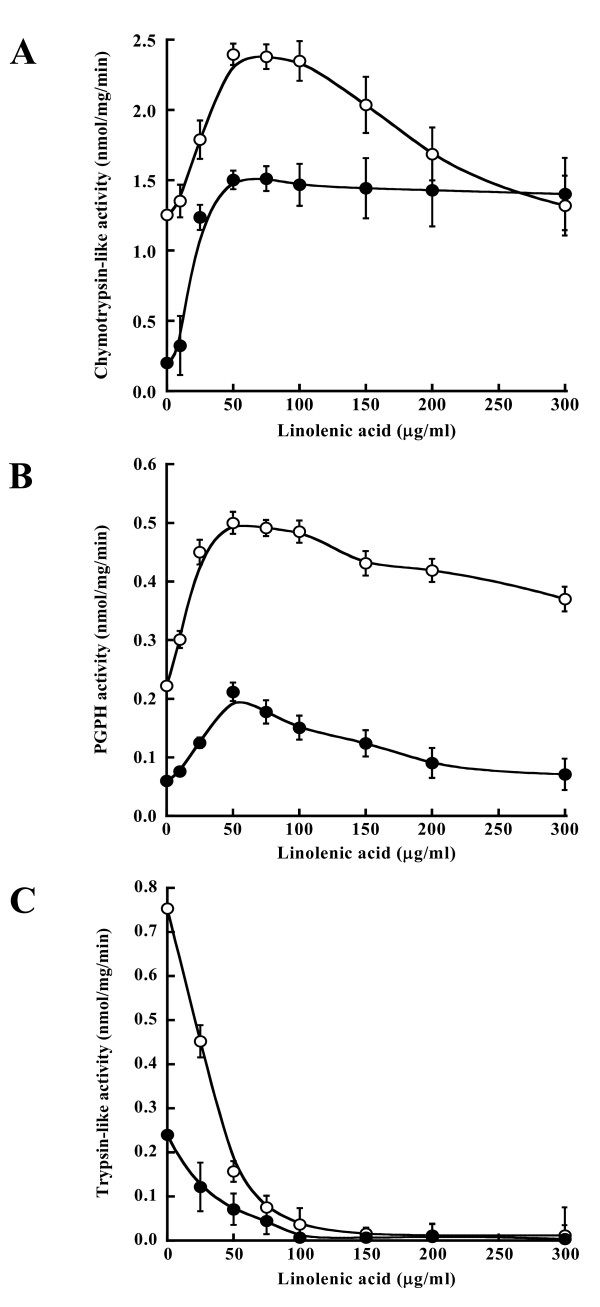
**Linolenic acid-mediated activation profile**. Assays were carried out in a 200 μl reaction mixture containing 50 mM Tris-HCl, pH 8.5, 1 μg/ml 20S proteasome, 1 mM EDTA-Tris, pH 8.5, 10% DMSO, and 10 μM peptidyl substrates (**A**: Suc-LLVY-MCA for chymotrypsin-like activity; **B**: Z-LLE-MCA for PGPH activity; **C**: Boc-LRR-MCA for trypsin-like activity) and increasing concentrations of linolenic acid at 37°C for 1 h. The reactions were started, stopped as described in methods. Symbols represent the wild-type strain (○) and the *rpt6-1 *mutant (●). Values are means ± SD of three independent experiments.

The polyunsaturated fatty acid, linolenic acid, is another well-known activator of the 20S proteasome [34] and its effect on hydrolysis of peptidyl substrates was also evaluated (Fig. 2). The obtained results were consistent with those of the SDS-mediated activation profile excluding optimal activity, i.e., the activities of the 20S_r _proteasome in the absence of linolenic acid were very low, but optimal activities were not found to converge with those of the 20S_w _proteasome, suggesting that the 20S_r _proteasome was unable to be fully stimulated by either linolenic acid or the experimental conditions. Our findings are similar to those of Thomas *et al*. [33] who reported linolenic acid-mediated stimulation of LLVY-activity and LLE-activity of the 20S proteasome from Ostrich skeletal muscle at pH 9.0, and inactivation of LRR-activity by the same acid. However, the linolenic acid-mediated optimal activities were found at ~50 μg/ml linolenic acid in both strains under the experimental conditions. This result distinguished the 20S_r _proteasome from the 20S_w _proteasome. Moreover, the same activity assay described above was carried out at 25°C under the same conditions in order to evaluate whether the low level of activities of the 20S_r _proteasome were altered by assay temperature. No significant changes of activities or activation profiles were detected (data not shown). This result was consistent with the possibility of a gating defect in the 20S_r _proteasome channel.

### Effect of reaction pH on peptidase activities of the 20S proteasome

**Figure 3:**
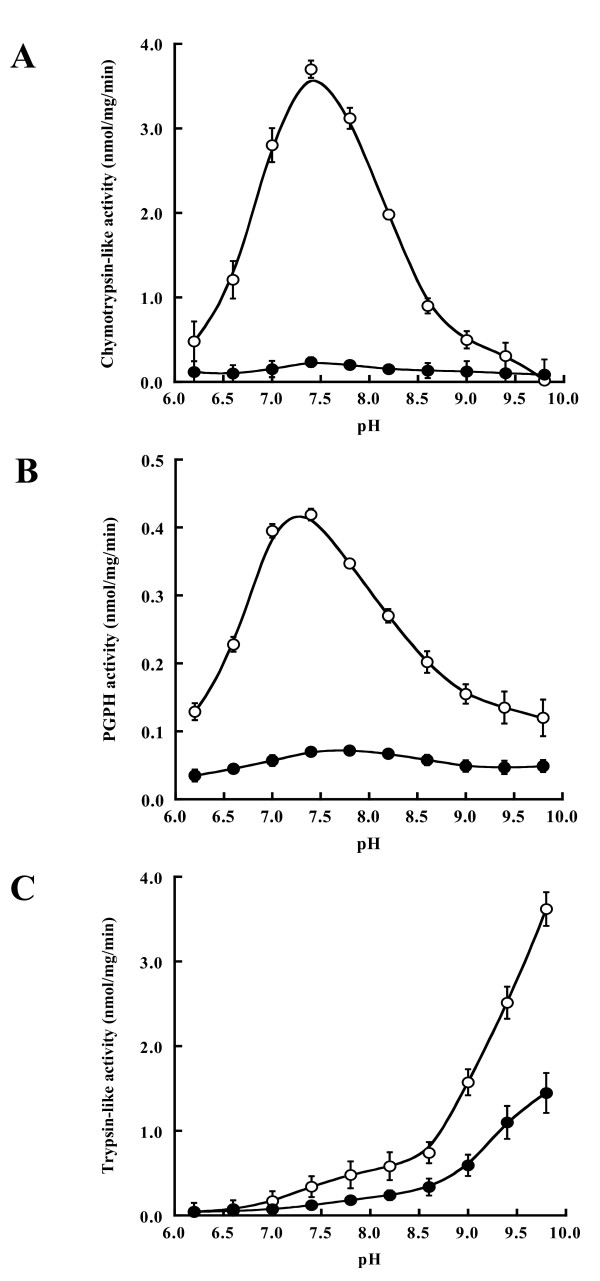
**The effect of reaction pH on peptidase activities**. Assays were carried out in a 200 μl reaction mixture containing 50 mM HEPES-Tris at pH 6.2–9.8, 1 μg/ml 20S proteasome, and 10 μM of peptidyl substrates (**A**: Suc-LLVY-MCA for chymotrypsin-like activity; **B**: Z-LLE-MCA for PGPH activity; **C**: Boc-LRR-MCA for trypsin-like activity) at 37°C for 1 h. Reactions were started and stopped as described in methods. Symbols represent the wild-type strain (○) and the *rpt6-1 *mutant (●). Values are means ± SD for three independent experiments.

In order to obtain clearer evidence for weak activity of the 20S_r _proteasome, we examined the effect of reaction pH on the peptide-hydrolyzing activities. No significant changes were found for 20S_r _proteasome activities over a wide range of pH values (Fig. 3). These results suggested that subunit composition of the 20S_r _proteasome might be related to the low activities. The LLVY- and LLE-activities of the 20S proteasome in both strains exhibited maximum values at neutral or weakly alkaline pH, whereas LRR-activities exhibited a maximum at alkaline pH. This observation is similar to the findings of Yanagawa *et al*. [35] who conducted a comparative study of the effect of reaction pH on three peptidase activities of the 20S proteasome from rice and spinach. Based on the low level of activities and the activation profiles, we concluded that the 20S proteasome in *rpt6-1 *likely had a gating defect.

### Subunit compositional analysis of the 20S proteasome by one-dimensional electrophoresis

**Figure 4:**
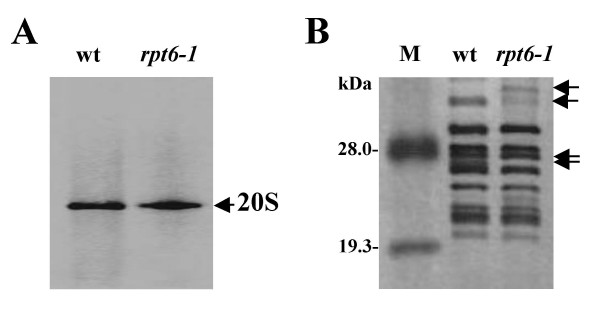
**Comparative subunit compositional analysis by one-dimensional electrophoresis**. (**A**) Purified 20S proteasomes were subjected to nondenaturing-PAGE in a 4% continuous polyacrylamide gel and (**B**) SDS-PAGE in a 12% polyacrylamide gel. The gels were stained with Coomassie Brilliant Blue R-250 and silver, respectively. The arrows indicate bands differing between strains.

Hoffman *et al*. [36] identified two electrophoretic forms of the 20S proteasome from rabbit reticulocytes, a slow form (20S_S_) and a fast form (20S_F_), and demonstrated the latter to be less active in the hydrolysis of the fluorogenic peptide Suc-LLVY-MCA. In this report, we conducted a comparative analysis of the migration of the intact 20S proteasome and its subunits by one-dimensional electrophoresis (Fig. 4) to determine whether the low activity of the 20S_r _proteasome was related to its mobility. Our data clearly demonstrated a distinct band in the nondenaturing 4% polyacrylamide gel without a significant change in mobility (Fig. 4A). But in SDS-PAGE, the banding pattern of the 20S_r _proteasome differed from that of the 20S_w _proteasome (Fig. 4B, arrows), suggesting that changes in the subunits in the 20S_r _proteasome were responsible for the low peptidase activities.

### Proteomic analysis and identification of subunits by mass spectrometry

**Figure 5:**
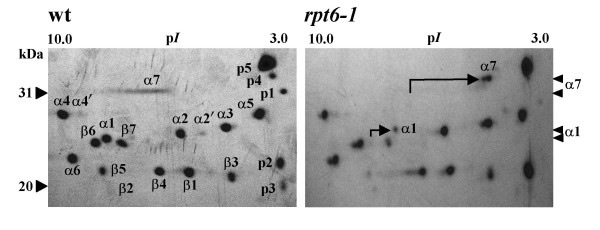
**Two-dimensional electrophoretic pattern of 20S proteasome subunits**. Purified 20S proteasomes (70 μg) from each strain were separated using a non-linear IPG strip (pH 3–10) in the first dimension (left to right) followed by SDS-PAGE in a 12% polyacrylamide gel in the second dimension (top to bottom). The 2-D gels were stained with Coomassie Brilliant Blue R-250. All labeled spots were identified by MALDI-TOF-MS and database searching (Table 1). The arrows indicate the shifts of the α1- and α7-subunits derived from the *rpt6-1 *proteasome towards the acidic side of the gel.

**Figure 6:**
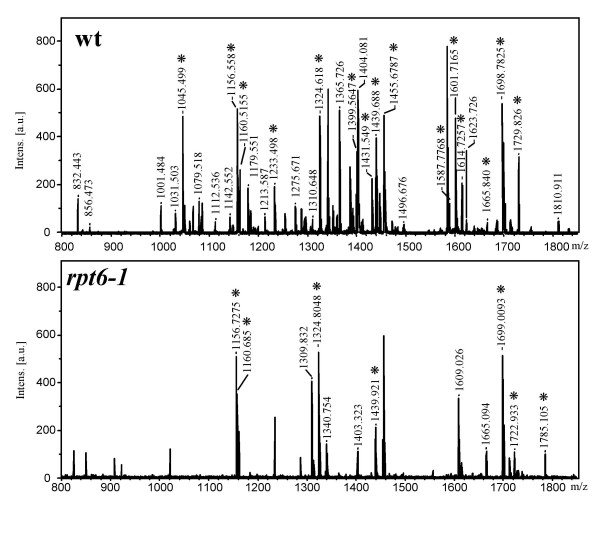
**Representative MS spectra of α1-subunits**. The trypsin-digested peptide mixture was extracted from gel pieces and analyzed by MALDI-TOF-MS. Peaks that matched in the w>database search indicated with asterisks. Panels correspond to a spot from the 20S_w _(**wt**) and 20S_r _(***rpt6-1***) proteasomes, respectively.

**Figure 7:**
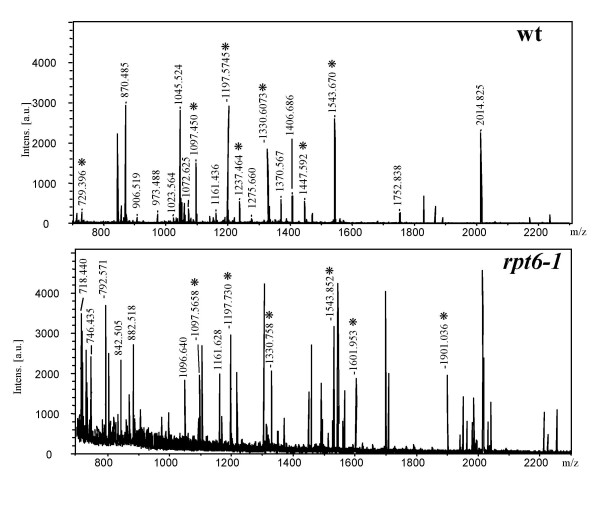
**Representative MS spectra of α7-subunits**. The trypsin-digested peptide mixture was extracted from gel pieces and analyzed by MALDI-TOF-MS. Peaks that matched in the w>database search are indicated with asterisks. Panels correspond to a spot from 20S_w _(**wt**) and 20S_r _(***rpt6-1***) proteasomes, respectively.

To test whether the observed low levels of activities of the 20S_r _proteasome were a consequence of the changes in subunit(s), we attempted to analyze each subunit in detail by two-dimensional electrophoresis (2-DE) followed by the sequence analysis of individual subunits extracted from the 2-D gel. The 2-DE revealed substantial similarities in spotting patterns of subunits of the 20S_r _proteasome to that of the wild-type with the exception of two spots (Fig. 5). All spots of the 20S proteasome separated by 2-DE were identified by MALDI-TOF-MS (Table 1). Among the fourteen subunits of the 20S proteasome, only the β2 subunit could not be identified due to insufficient protein on the gel. Two α-subunits (α1, α7) in *rpt6-1 *were found to differ from their wild-type counterparts in spotting pattern in both p*I *and molecular weight (Fig. 5). Representative PMF maps of the α1- and α7-subunits of both strains are shown in figures 6 and 7, respectively. In *rpt6-1*, the α1-subunit shifted slightly and the α7-subunit shifted markedly towards the acidic side of the gel, while both migrated slowly in SDS-PAGE (Figs. 5 and 4B) indicating that the weak activities of the 20S_r _proteasome are a consequence of changes in these two α-subunits. At least two explanations can be offered to account for the lower activity of the 20S proteasome in the *rpt6-1 *mutant. One, the *rpt6-1 *mutation caused changes in the α-subunits of the 20S proteasome by interfering with assembly of the 26S proteasome. Two, stress caused by the *rpt6-1 *mutation induced subsequent compensatory changes in the subunits.

**Table 1:** A list of 20S proteasome subunits identified by peptide mass fingerprinting.

Spot no.	Protein description	Accession no. (gi)	Theoretical mass (M_r_)	Theoretical p*I*	Matched peptide	Sequence coverage (%)
20S subunits	α1	6321427	27983	5.89	15	48
	α2	6323547	27145	5.52	16	40
	α3	3114271	27003	5.71	16	45
	α4	3114272	26853	6.04	17	54
	α5	3114273	26497	4.80	14	43
	α6	3114274	25457	7.08	17	61
	α7	14488811	31386	5.04	12	32
	β1	11513426	21709	5.36	14	60
	β2					
	β3	3114278	22459	5.05	7	32
	β4	6320849	22503	5.83	9	41
	β5	3114280	23314	5.94	8	32
	β6	3114281	25457	7.08	17	61
	β7	3114282	25903	5.75	9	41

Other proteins						
p1	SEC65	671640	31150	9.14	19	37
p2	YCL012w	10383775	25809	4.84	10	24
p3	YDR179c	45269225	19464	5.07	8	27
p4	(Putative protein of unknown function)	6321353	32092	10.02	7	15
p5	Sec17	6137605	32764	4.96	4	17

The top BLAST hit for each spot, its accession number, and sequence coverage to the *S. cerevisiae* proteasomal subunit are shown. The peptide mass fingerprinting was performed using the protein spots from the wild-type strain as shown in Figure 5. The differentially spotted proteins were identified in both strains.

Groll *et al*. [31] demonstrated that the channel of the 20S proteasome is too narrow for substrate entry, but that the proteasome may become active when the channel is open. As shown in their study, activity of the 20S proteasome is controlled by the α-channel. Here, we identified two high molecular weight forms of the α-subunits in *rpt6-1 *that resulted in weak 20S_r _proteasome activity. We speculate that the α-subunit(s) are differentially modified in the 20S_r _proteasome and block the gated channel that allows restricted entry of substrate into the proteasome catalytic cavity since the substrate specific activities in the absence of SDS were low, but optimal activities in the presence of SDS were found to converge with those of the 20S_w _proteasome (Fig. 1).

**Figure 8:**
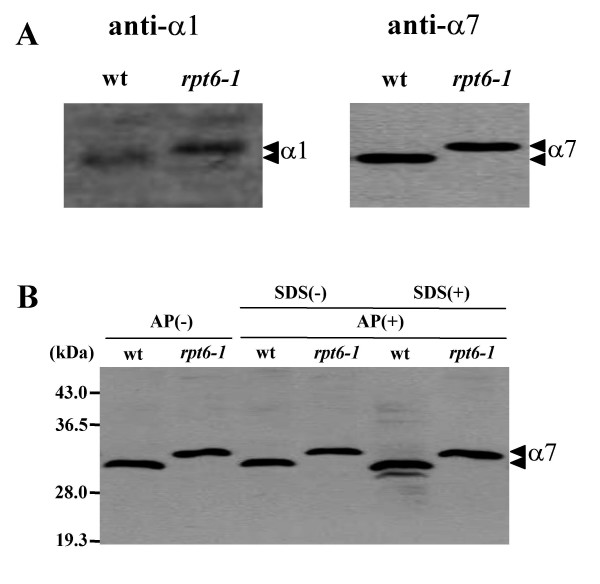
**Identification of α1- and α7-subunits by western blot analysis**. (**A**) The 20S proteasomes were subjected to SDS-PAGE in a 12% polyacrylamide gel and electrophoretically transferred to a PVDF membrane. The α1- and α7-subunits were detected using their respective antibodies as described in the methods. (**B**) Cells from wild-type and the *rpt6-1 *mutant (wt, *rpt6-1*) grown at 25°C (OD_600 nm _= 1.0) were harvested and the cell extracts were applied to a Q-sepharose column. Active Q-sepharose fractions were concentrated and treated with (AP+) or without (AP-) alkaline phosphatase as described in the methods. The samples were then subjected to SDS-PAGE in a 12% polyacrylamide gel. The α7-subunit was detected by immunostaining with anti-α7 antibody [26,27].

To reconfirm the results described above, we characterized the differentially migrated subunits in the 20S_r _proteasome by Western blotting. The results reconfirmed the slow migration of α1 and α7 in the 20S_r _proteasome compared to those of the 20S_w _proteasome (Fig. 8A). Phosphorylation of the proteasome has been reported to modulate a number of functions including catalytic activity [37]. Iwafune *et al*. [26,38] demonstrated that the α7-subunit in yeast is a major phosphorylatable subunit. Regulation of the proteasome complex by phosphorylation of the α7-subunit in COS-7 cells has been demonstrated by Rivett and colleagues [39,40]. Tokumoto *et al*. [27] detected a high molecular weight phosphorylated α4-subunit in the 26S proteasome from immature *Xenopus *oocyte. These findings led us to test whether the differentially migrating α-subunits in *rpt6-1 *were phosphorylated. No significant change in mobility or intensity was detected for α7 treated with alkaline phosphatase (Fig. 8B) or for the similarly treated α1-subunit (data not shown).

**Figure 9:**
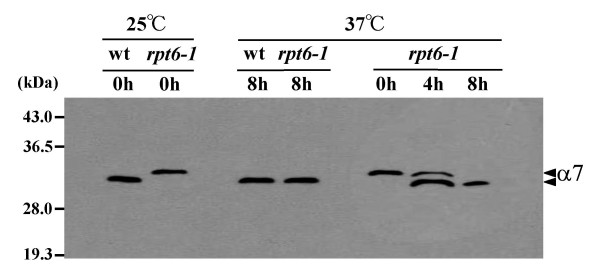
**Molecular weight level of the α7-subunit in the *rpt6-1 *mutant under permissive and restrictive conditions**. Cells from wild-type and the *rpt6-1 *mutant (wt, *rpt6-1*) were grown at 25°C to exponential phase at OD_600 nm _= 2.0 (0 h). The cultures were then immediately shifted to 37°C and incubated for several hours as indicated. Cell extracts were applied to a Q-sepharose column. The molecular weight level of the α7-subunits in both strains produced during the indicated incubation period was assayed by Western blotting using anti-α7 antibody.

The *rpt6-1 *mutant arrests cell division within one cycle at the restrictive temperature [13] and under normal growth conditions, we found changes in the α1- and α7-subunits of the 20S proteasome in the *rpt6-1 *mutant. One question that remains to be clarified is whether the observed changes are affected at the restrictive temperature. The data presented in figure 9 indicate that the change in α7 in the *rpt6-1 *mutant grown at 25°C disappeared during the response to cell stress. Interestingly, in parallel with the disappearance, normalization of LLVY-hydrolyzing activity was observed in the absence of SDS (Table 2). One likely explanation of the data presented here is that the α1- and α7-subunits exist in the 20S_r _proteasome in an immature form that result in a gating defect in the proteasome channel. A qualitative change to these subunits occurred at 37°C to cause them to mature because under stressful conditions, the cell is in greatest need of optimal proteasome function. Alternatively, newly synthesized proteasomes in the *rpt6-1 *mutant may be refractory to modification due to excessive stress at the restrictive temperature.

**Table 2:** Chymotrypsin-like activity of the 20S proteasome in the *rpt6-1 *mutant as a function of temperature shift^*a*^.

Incubation temp.	Chymotrypsin-like activity (nmol/mg/min)		wt/*rpt6-1*	Chymotrypsin-like activity (nmol/mg/min)		wt/*rpt6-1*
	SDS (-)			SDS (+)		
				
	wt	*rpt6-1*		wt	*rpt6-1*	
25°C	0.58 ± 0.02	0.07 ± 0.03	8.0	4.42 ± 0.29	2.63 ± 0.31	1.6
37°C	0.42 ± 0.04	0.51 ± 0.04	0.8	3.33 ± 0.70	3.73 ± 0.22	0.8

^*a*^Exponentially-growing cells at 25°C were shifted to 37°C and incubated for 8 h. Activity assays were carried out with the active Q-sepharose fractions from both strains containing equal amounts of proteasome as described in the methods using Suc-LLVY-MCA as a substrate in the presence (0.01%) and absence of SDS. The values are means ± SD of three independent experiments.

Although the purified proteasomes from both strains produced single bands in nondenaturing-PAGE (Fig. 4A), they differed in composition in 2-DE (Fig. 5). One possibility is that the compositional differences reflect differences in co-purified proteasome-interacting proteins (PIPs). Five proteins (p1, p2, p3, p4, p5) found with the 20S proteasome were also identified by database searching (Table 1). The protein, p1 was found in the wild-type strain but not in the *rpt6-1 *mutant. Therefore, we cannot rule out the possibility that the PIP, p1, plays a supplementary role in normal 20S proteasome function in yeast.

To show that the changes found in the 20S_r _proteasome were dependent on the *rpt6-1 *mutation, the *rpt6-1 *strain was transformed with the wild-type allele as described in methods. The *RPT6 *allele was found to complement the temperature sensitivity of the mutant. The 20S proteasome purified from the transformant was analyzed to examine whether changes in the α-subunits and the weak peptidase activities were eliminated. Interestingly, the banding pattern of the 20S proteasome subunits of the transformant was found to be similar to that of wild-type by SDS-PAGE analysis using a 12% polyacrylamide gel. Further, the slow mobility of the α-subunits and the weak peptidase activities were rescued in the transformant as well (data not shown).

## Conclusion

The 20S proteasome purified from the *rpt6-1 *mutant exhibited low levels of peptidase activities compared to the wild-type strain. The activator-mediated activation profile of the 20S proteasome from *rpt6-1 *also differed from that observed in the wild-type 20S proteasome. The α1- and α7-subunits of the 20S proteasome in the *rpt6-1 *mutant were found to differ from their wild-type counterparts.

## Abbreviations

SDS: Sodium dodecylsulfate; TFA: Trifluoroacetic acid; p*I*: Isoelectric point; PGPH: Post-glutamylpeptide hydrolysis; IPG: Immobilized pH gradient; PMF: Peptide mass fingerprint

## Authors' contributions

AC carried out the biochemical and molecular experiments, the peptide mass fingerprinting, and drafted the manuscript. TT participated in design and coordination of experiments and helped to draft the manuscript. HD helped to perform the peptide mass fingerprinting. TU helped to construct plasmids. SY assisted with the design and coordination of the study. All authors read and approved the final manuscript.
